# Microfluidic Droplet Digital PCR Is a Powerful Tool for Detection of BRAF and TERT Mutations in Papillary Thyroid Carcinomas

**DOI:** 10.3390/cancers11121916

**Published:** 2019-12-02

**Authors:** Dorina Ylli, Aneeta Patel, Kirk Jensen, Zhao-Zhang Li, Maria Cecilia Mendonca-Torres, John Costello, Cristiane Jeyce Gomes-Lima, Leonard Wartofsky, Kenneth Dale Burman, Vasyl V. Vasko

**Affiliations:** 1 Thyroid Cancer Research Center, MedStar Health Research Institute, 100 Irving St NW, Washington, DC 2010, USA; dorina.ylli@umed.edu.al (D.Y.); Cristiane.Lima@medstar.net (C.J.G.-L.); leonard.wartofsky@medstar.net (L.W.); Kenneth.D.Burman@medstar.net (K.D.B.); 2 Division of Endocrinology, Department of Internal Medicine, MedStar Washington Hospital Center, 110 Irving St NW, Washington, DC 2010, USA; 3 Department of Imaging and Clinical Semeiotic, Faculty of Medicine, University of Medicine Tirana, 371 Dibra St, 1005 Tirana, Albania; 4 Department of Pediatrics, Uniformed Services University of the Health Sciences, 4301 Jones Bridge, Bethesda, MD 20814, USA; aneeta.patel@usuhs.edu (A.P.); kirk.jensen@usuhs.edu (K.J.); maria.mendonca-torres@usuhs.edu (M.C.M.-T.); john.costello.ctr@usuhs.edu (J.C.); 5 Biomedical instrumentation center, Uniformed Services University of the Health Sciences, 4301 Jones Bridge, Bethesda, MD 20814, USA; zhaozhang.li@usuhs.edu

**Keywords:** thyroid cancer, digital PCR, oncogenes, quantification, metastasis, BRAF, TERT

## Abstract

We examined the utility of microfluidic digital PCR (dPCR) for detection of *BRAF* and *TERT* mutations in thyroid tumors. DNA extracted from 100 thyroid tumors (10 follicular adenomas, 10 follicular cancers, 5 medullary cancers, and 75 papillary thyroid cancer (PTC) were used for detection of *BRAF* and *TERT* mutations. Digital PCRs were performed using rare mutation SNP genotyping assays on QuantStudio 3D platform. In PTCs, *BRAF*V600E was detected by dPCR and Sanger sequencing in 42/75 (56%) and in 37/75 (49%), respectively. *BRAF*V600E was not detected in other tumors. The ratio of mutant/total *BRAF* alleles varied from 4.7% to 47.5%. These ratios were higher in classical PTCs (27.1%) as compared to follicular variant PTCs (9.4%) *p* = 0.001. In PTCs with and without metastases, the ratios of mutant/total *BRAF* alleles were 27.6% and 18.4%, respectively, (*p* = 0.03). In metastatic lesions percentages of mutant/total BRAF alleles were similar to those detected in primary tumors. *TERT*C228T and *TERT*C250T were found in two and one cases, respectively, and these tumors concomitantly harbored *BRAF*V600E. These tumors exhibited gross extra-thyroidal extension, metastases to lymph nodes, and pulmonary metastases (one case). Our results showed that dPCR allows quantitative assessment of druggable targets in PTCs and could be helpful in a molecular-based stratification of prognosis in patients with thyroid cancer.

## 1. Introduction

Papillary thyroid cancer (PTC) is the most common endocrine malignancy and is also increasing at the fastest incidence rate of any malignancy [1]. Extensive characterization of the mutational landscape of thyroid cancer, which has accelerated over the past few years after the introduction of next-generation sequencing, has provided a basis for creating multigene mutational panels for the detection of cancer in thyroid nodules [2,3]. Somatic alterations of genes involved in the mitogen-activated protein kinase (MAPK) pathway were frequently detected in PTC, with RET (rearranged during transfection) rearrangements accounting for 10–15%, Ras point mutations (rat sarcoma viral oncogene homolog) for 10–15%, and *BRAF* (B-type Raf kinase) point mutations comprising the majority of 40–60% of all PTC more than 1 cm, and 60–75% of those with known mutations [3,4,5].

*BRAF* is a cytoplasmic serine-threonine protein kinase and among the three forms of RAF kinases, *BRAF*, is the most potent activator of the MAPK pathway [6,7]. The vast majority of BRAF alterations are characterized by a single amino acid substitution of valine by glutamic acid in a mutational hotspot at amino acid position 600 (BRAF c.1799T>A (p.Val600Glu), referred to hereafter as *BRAFV600E*). This exchange mimics the phosphorylation of amino acid residues T599 and S602 and induces a conformational change of the activation segment leading to a constitutive kinase activity of BRAF and phosphorylation of downstream targets [8].

The oncogenic and transforming function of the mutated *BRAFV600E* has been demonstrated in thyroid cells. *BRAFV600E* has been shown to initiate thyroid follicular cell transformation both in culture and in transgenic mice [9]. The *BRAFV600E* mutation showed a high specificity for PTC, especially the classic variant, whereas it was never found in follicular and medullary thyroid carcinoma or in benign thyroid neoplasms [10]. It was also detected in 13.9–25% of anaplastic thyroid carcinomas, most likely originating from the dedifferentiation of PTC [11,12,13,14,15]. From 29 studies reporting on *BRAF* mutations in more than 2,000 examined thyroid cancers, the average frequency of mutations in PTC was 44% and in anaplastic thyroid cancer (ATC) was 24% [14].

Previous studies demonstrated a relationship between the presence of the *BRAFV600E* mutation and more aggressive clinical and pathological features of PTCs [16,17]. Interestingly, more aggressive behavior of *BRAFV600E* positive PTC was also reported in small tumors. Papillary thyroid micro-carcinomas are generally indolent, however, *BRAFV600E* positive micro-carcinomas are associated with extrathyroidal extension and lymph node metastasis [17]. In advanced PTCs, *BRAFV600E* mutations were noted to be at an increased frequency (62%) in recurrent and/or metastatic tumors from iodine-refractory PTC patients [18,19]. In this context, detection of *BRAFV600E* in primary tumors has been proposed as a marker predicting the status of iodine uptake in case of distant metastases as the mutation was associated with non-radioiodine-avid status in PTC [20]. Large multicenter studies have demonstrated an association between *BRAF* V600EE and PTC recurrence as well as PTC-specific mortality [21]. A greater percentage of *BRAF* V600EE patients were diagnosed at a higher stage of cancer, suggesting a faster and more aggressive growth pattern compared to the mutation negative patients, and the higher stage accounted reflected in higher death rate [21]. However, given the high prevalence of *BRAFV600E*, it was suggested that detection of *BRAFV600E* may not be practical to generally recommend aggressive treatment for all *BRAF* mutation-positive PTC.

Interestingly, coexisting *BRAFV600E* and *TERT* (Telomerase Reverse Transcriptase) promoter mutations were shown to be particularly associated with high-risk clinico-pathological characteristics of PTC, and PTC-specific mortality [22,23,24]. Two mutually exclusive *TERT* promoter mutations (TERT: c-124C> T (C228T) and c-146C> T (C250T) referred hereafter as C228T and C250T) have been reported in PTCs and ATCs [22,23,24]. Molecular analysis of 144 cases of ATC revealed the presence of *TERT* promoter mutations (C228T and C250T) in 54% of examined cases [15]. *TERT* encodes the catalytic subunit of telomerase, the enzyme responsible for extending telomeres and thereby preventing replicative senescence. These mutations confer the *TERT* promoter increased transcriptional activities. It was proposed that the MAPK pathway could promote the expression of TERT through upregulating the E-twenty-six (ETS) factors [25,26]. Indeed, coexistence of *BRAF* V600E and *TERT* promoter mutations was shown to be associated with increased expression of TERT in thyroid cancer. These data provided a molecular mechanism explaining the strong synergism between *BRAFV600E* and *TERT* promoter mutations in promoting the mortality of PTC.

Given the utility in knowing the status of *BRAFV600E* and *TERT* mutations in thyroid cancer, different methods have been used to access the mutation status. Sanger sequencing, allele-specific amplification PCR (ASA-PCR), quantitative PCR (qPCR), pyrosequencing, and next generation sequencing are some of the techniques used [27,28,29]. Different molecular diagnostic approaches had different sensitivity for the detection of *BRAFV600E* mutations in PTC [30]. Recent study demonstrated that using sequencing, *BRAFV600E* mutations were detected in 37% of patients; however, when ASA-PCR and qPCR technologies were used *BRAFV600E* mutations were found in 57% and 60% of patients, respectively. It has been also shown that DNA quality had a significant impact on results of *BRAFV600E* testing. Thus, applying methods with different sensitivities to the detection of *BRAFV600E* mutations may result in different results for the same patient; such data can influence stratification of patients into different risk groups, leading to alteration of treatment and follow-up schemes.

Recent studies demonstrated that molecular analysis of DNA using droplet digital (d)PCR technique has advantages as compared to Sanger sequencing or real time PCR approaches [29,31,32]. Digital PCR (dPCR) analysis of DNA appears particularly attractive for patients with thyroid cancer, a tumor characterized by a high frequency of hotspot mutations in *BRAFV600E*. However, currently there are limited data demonstrating the clinical utility of the dPCR-based detection of mutations in patients with thyroid tumors [33]. Beside the high sensitivity, dPCR allows a quantification of the mutant allele, therefore providing a deeper insight into the relationships between the mutation status and tumor’s clinico-pathological characteristics, and response to therapy.

The aim of this study was to determine the utility of digital PCR technique in detecting *BRAFV600E* and *TERT* promoter mutations (C228T and C250T) in thyroid cancer tissue samples. We also sought to establish whether the quantitative evaluation of *BRAFV600E* and *TERT* promoter mutations could be comparable with data obtained by Sanger sequencing.

## 2. Results

### 2.1. Optimization of dPCR for Detection of BRAFV600E and TERT Promoter Mutations

The optimization of dPCR conditions for detection of mutations was performed in accordance with guidelines for performing dPCR experiments [34,35]. The primer sets were tested for amplification of the genomic region of interest by end-point PCR. Amplification conditions were optimized by testing a range of annealing temperatures (50–65 °C), and optimal amplifications for *BRAFV600E*, *TERTC228T*, and *TERTC250T* were achieved at 60 °C, 55 °C, and 55 °C, respectively. Next, digital PCRs were performed at a gradient of annealing temperatures ranging from 50 °C to 65 °C, and optimal segregations for *BRAFV600E*, *TERTC228T*, and *TERTC250T* were at 60 °C, 55 °C, and 55 °C, respectively. Hereafter, all subsequent dPCR assays were performed with established annealing temperatures.

For each experiment in this project, the chip quality control was performed. The software assessed whether the data on a chip were reliable based upon loading, signal, and noise characteristics and displayed quality indicators for each chip in a project. Subsequent data analysis was performed using the cloud-based QuantStudio 3D Analysis Suite software in the absolute quantification module maintaining automatic settings. For each dPCR experiment, the mean number of copies per partition and the number of estimated copies per total reaction volume for the unknown samples were calculated. For each run, at least one negative template control (NTC), wild type positive control and rare mutation positive controls were included. The sensitivity, minimum rare positive points, average false positives, and standard deviation of false positives were calculated for each experimental dPCR run by Analysis Suite software.

We calculated the limit of blank (LOB), as well as the limit of detection (LOD). The LOB was calculated based on the replicates of the experiments when no mutant analyte was tested by using the following equation: LOB= mean_blank_ + 1.645 (SD_blank_). Results are presented as the number of false positive partitions to the number of the total partitions per experiment. For *BRAFV600E*, *TERT*C228T, and *TERT*C250T the LOB was: 0.0093%, 0.01%, and 0.07% respectively. Similarly, we used the LOB to calculate the LOD, which is the lowest analyte partition likely to reliably distinguish from LOB and at which detection is feasible. The equation we used to calculate the LOD is LOD= LOB + 1.645 (SD _low concentration sample_). For *BRAFV600E*, *TERT*C228T, and *TERT*C250T the LOD was: 0.04%, 0.09%, and 0.13% respectively.

### 2.2. Detection of BRAFV600 and TERT Promoter Mutations in Thyroid Cancer Cell Lines

**Figure 1:**
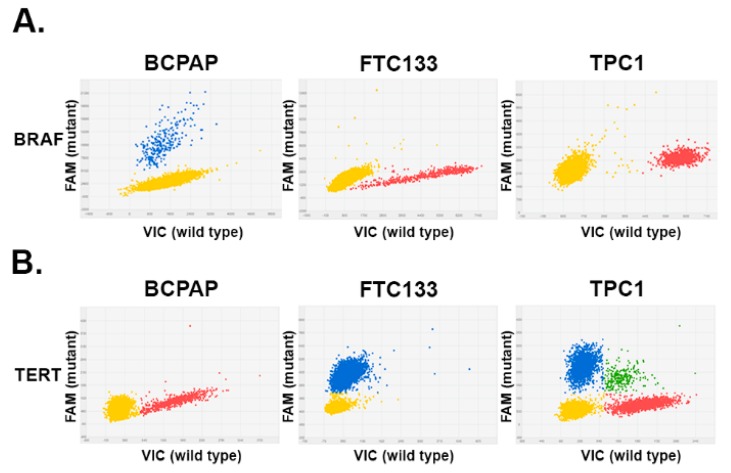
Detection of BRAF (B-type Raf kinase) and TERT (Telomerase Reverse Transcriptase) mutations by digital PCR in thyroid cancer cell lines. Each panel represents a single dPCR experiment whereby a DNA sample was segregated into individual wells and assessed for the presence of the mutant allele and wild type allele using two different fluorophores (6-Carboxyfluorescein (FAM) and 2′-chloro-7′phenyl-1,4-dichloro-6-carboxy-fluorescein (VIC). The signals from the FAM (blue) and VIC (red) dyes were plotted on the *y*-axis and *x*-axis, respectively. The yellow cluster represented unamplified wells (negative calls). (**A**) 2D plots of dPCR reads out of DNA extracted from thyroid cancer cells. Blue cluster represented wells that were positive for the BRAFV600 mutation. (**B**) Results of dPCR analysis of TERT228 analysis in thyroid cancer cells, demonstrating the presence of mutant (blue) and wild type (red) alleles. Green cluster represents wells containing both VIC and FAM dyes.

We next examined *BRAFV600E* copy number status in thyroid cancer cell lines with known *BRAFV600E* mutation status (*BRAFV600E* positive BCPAP, and *BRAFV600E*-negative FTC133 cells and TPC1 cells). As demonstrated in Figure 1A, mutant copies (blue dots) were detected in DNA extracted from BCPAP cells. In contrast, only wild type *BRAF* copies were detected in FTC133 and TPC1 cells.

Mutant *BRAF* alleles were also accurately detected in diluted mixtures of mutant and wild type *BRAF* at ratios of 1:1, 1:10, 1:100, and 1:1000. The 1:1000 mix contained only 0.01 ng of mutant *BRAF*. Microfluidic dPCR, but not RT PCR or Sanger sequencing, allowed detection of mutant copies in a mixture of 9.99 ng of DNA from FTC133 cells with 0.01 ng of DNA from BCPAP cells.

The same approach was employed to establish dPCR conditions for detection of *TERTC228T* and *TERTC250T* mutations. Mutations *TERTC228T* were detected in FTC133 and TPC1 thyroid cancer cells, but not in BCPAP cells (Figure 1B). Mutations *TERTC250T* were not detected in examined thyroid cancer cells.

### 2.3. Detection of BRAFV600 and TERT228 in Follicular Adenomas, Follicular Cancers, and Medullary Thyroid Cancers

After establishing experimental conditions for detection of *BRAFV600E* and *TERT* promoter mutations, we next examined DNA extracted from human thyroid tissue samples. Taking into consideration high sensitivity of dPCR we initially examined set of thyroid tumors that have been shown to be negative for BRAFV600 or TERT228 mutations. Analysis of DNA extracted from 10 follicular adenomas (FA), 10 follicular cancer (FC), and 5 medullary thyroid cancer (MTC) showed no presence of BRAFV600 or TERT228 alleles. In all experiments, DNA from BCPAP and TPC1 cells were used as positive controls for detection of BRAFV600 and TERT228.

### 2.4. Detection of BRAFV600 by Digital PCR in Papillary Thyroid Cancers

Normal thyroid tissue adjacent to the tumor was available for analysis in 23/75 patients with PTCs. Analysis of BRAF in these samples revealed only wild type alleles in 22/23 samples. In one case, mutant BRAFV600 alleles were detected in normal thyroid, and the mutant/total ratio was 1.4%.

In PTCs tissue samples, BRAFV600 was detected in 42/75 (56%) cases, and the ratio of mutant/total BRAF alleles ranged from 4.7% to 47.5% (average 25.9%). Sanger sequencing confirmed the presence BRAFV600 in 37 cases, with all PTCs having a ratio of mutant/total BRAF alleles below 10% being undetected by Sanger sequencing.

Analysis of BRAFV600 mutation in function of clinico-pathological characteristics of examined PTCs is presented in Table 1. BRAFV600 was more frequently detected in classic papillary thyroid cancer (CPTC) as compared to follicular variant papillary thyroid cancer (FVPTC). Also, BRAFV600 mutation was associated with presence of extra-thyroidal invasion and presence of lymph node metastases at the time of surgery.

**Figure 2:**
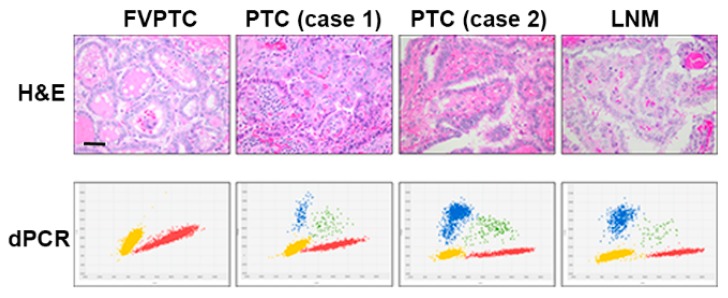
Correlation between percentages of BRAF mutant alleles with papillary thyroid cancers (PTCs) morphology. Top panel demonstrates morphological features of examined tumors, and bottom panel shows results of dPCR analysis. In follicular variant papillary thyroid cancer (FVPTC), classic papillary thyroid cancer (CPTC) with lymphocytic infiltration of stroma (case 1), CPTC (case 2), and in metastatic lesion (LNM), the percentages of mutant/total BRAF were 0%, 10.6%, 42.6%, and 40.1%, respectively. Scale bars 100 µm; /magnification: 400×.

Quantification of BRAFV600 alleles in PTC tissue showed that the percentage of mutant/total alleles was significantly lower in FVPTC (9.4%) compared to CPTC (27.1%). As demonstrated in Figure 2, a low percentage of the mutant BRAF alleles was also detected in DNA extracted from CPTC with lymphocytic infiltration in tumor stroma.

The results of BRAF quantitative analysis and pathological characteristics are summarized in Table 2. The quantitative analysis of BRAF mutant alleles showed a significant difference between PTCs presenting with lymph node metastases as compared to PTCs without metastases (27.6% vs. 18.4% respectively; *p* = 0.03). No significant difference was observed between males and females (males 28.72% vs. females 25.28%, *p* = 0.48).

The quantification of mutant BRAF alleles in primary PTCs and in corresponding metastatic lesions was performed in 11 cases (all CPTCs). There were no statistically significant differences in percentage of mutant alleles, in primary PCT and corresponding lymph node metastases the ratios mutant/total BRAF alleles were 29% and 24.2%, respectively (*p* = 0.34).

### 2.5. Detection of TERT228 and TERTC250T by Digital PCR in Papillary Thyroid Cancers

**Figure 3:**
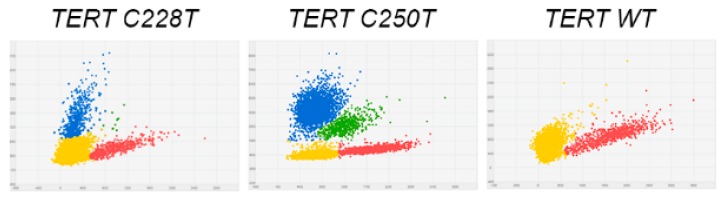
Results of TERT228 and TERTC250T analysis in human papillary thyroid cancer. 2D plots of dPCR reads out of DNA extracted from classical PTC. DNA extracted from these tumors contained mutant (blue) and wild type (red) TERT alleles.

In PTCs tissue samples, TERT228 was detected in 2/75 (2.6%) cases (Figure 3). Both TERT228 positive tumors were CPTCs and, in addition to TERT228 harbored BRAFV600. Two patients with TERT228 positive tumors were 63 and 68 years of age, and had large tumors (5 and 7 cm). These PTCs exhibited morphological features of aggressive thyroid cancers (gross extra-thyroidal extension, metastases to lymph nodes of central and lateral compartments). One patient presented with pulmonary metastases at time of surgery. In both cases, tissues from adjacent to tumor normal thyroid tissue, as well as lymph node metastases were available for analysis. In normal thyroid tissue samples, TERT228 were not detectable. The quantitative assessment of TERT228 in primary tumors and in corresponding metastatic lesions showed increased in the ratio of mutant/total TERT alleles in metastases as compared to primary tumors. In case 1, the percentages of TERT228 in primary and metastases were 29.8% and 41.3%, respectively. In case 2, the percentages of TERT228 in primary and metastases were 44.6% and 48.7%, respectively.

## 3. Discussion

This study examined the utility of microfluidic digital PCR for analysis of *BRAFV600E* and *TERTC228T* mutations in thyroid papillary cancers. We first focused on detection of *BRAFV600E* in thyroid cancer tissue samples because numerous studies demonstrated that pharmacological targeting of *BRAFV600E* represents a new therapeutic approach for patients with thyroid cancer. Moreover, the availability of already approved *BRAF* inhibitors offers an opportunity to employ this treatment strategy in case of *BRAF*-positive progressive thyroid cancers [36]. Recent studies demonstrated that treatments with *BRAF* inhibitor dabrafenib stimulated radioiodine uptake in patients with metastatic *BRAFV600E*-positive iodine-refractory PTCs [37]. In addition, another *BRAF* inhibitor, vemurafenib, showed anti-tumor activity in *BRAFV600E*-positive PTCs refractory to radioactive iodine [38]. We assumed that, in this context, information on *BRAF* mutation status in thyroid cancer tissue could be important in the determination of treatment strategy.

Second, we examined *TERT* mutations in patients with thyroid tumors. The increase in mutation burden and the presence of both *BRAF* and *TERT* promoter mutations has been shown to be useful in assessing risk stratification in patients with PTCs, as the presence of both mutations have been associated with a poorer prognosis [39]. Recent study examined prognostic value of *BRAFV600E* and *TERT* mutations in PTC-related mortality in a large cohort of PTCs patients (1051 patients). This study demonstrated that mortality for cases with both mutations remained significant (hazard ratio (HR), 9.34; 95% confidence interval (CI), 2.53–34.48) after adjustment for clinicopathological factors, and the genetic duet showed a strong incremental and synergistic impact over either mutation alone [40].

We thought to determine the utility of the microfluidic digital PCR technique for assessment of *BRAF* and *TERT* mutations in routinely processed thyroid tissue samples, with the aim to provide ‘on-time’ information for the clinical thyroidologists regarding possible treatment options, as well as data informing the prognosis in patients with thyroid cancer.

Digital methods have been shown to improve specificity and sensitivity of mutation detection through separation of template molecules into individual reaction vessels with approaches using either microfluidics, oil emulsion, or by combining microfluidics and emulsion PCR. Microfluidic digital PCR was shown to be reliable in distinguishing mutant from wild type alleles with no false positive results in cancer patients [41].

Using this technique, we detected *BRAFV600E* in 56% of the PTCs, with no false positive results, and sensitivity of dPCR was higher as compared to Sanger sequencing. These findings are consistent with previous studies demonstrating that PCR techniques (ASA-PCR and qPCR) had higher sensitivity for detection of *BRAFV600E* as compared to Sanger sequencing [30,31]. Our study also confirmed that application of methods with different sensitivities to the detection of mutations may result in different results for the same patient.

*BRAFV600E* was detected more frequently in CPTC as compared to FVPTC, which is in accordance with previously reported findings [10,11,21,24]. The quantitative evaluation of mutant/total *BRAF* alleles showed a spectrum varying from 4.7% to 48%. The quantification of *BRAF* mutation in PTC using pyrosequencing showed similar variability, with reported range varying from 8% to 41% [42]. The differences in *BRAFV600E* percentages could be attributed to different tumor’s purity and different abundance of epithelial cancer cells in examined tissue samples. The presence in the analyzed specimen of non-cancer cells-such as lymphocytes, endothelial cells, and fibroblasts-could provide a possible explanation. However, authors who have used micro dissection (selecting only cancer cells) or have corrected for the amount of non-cancer cells can still observe an inter- and intra-tumor variations [43,44]. It was also suggested that *BRAF* mutation in PTC is a late sub-clonal event, and the same tumor can contain epithelial cells with wild type *BRAF* as well as cells with mutant *BRAF* [45].

The percentage of *BRAF* mutant alleles was higher in PTCs presenting with lymph node metastases at the time of surgery, and *BRAFV600E* were detected in metastatic lesions. These observations are in agreement with previous study showing that lymph node metastases were more frequent in PTCs with a high (≥20%) abundance of mutant alleles than in those with a low abundance of mutant alleles [42]. These results support the potential role of BRAF in thyroid cancer progression, and suggest that quantitative analysis of the *BRAF* mutation could provide additional information regarding thyroid cancer propensity to develop metastases.

Different methods have been previously proposed for the detection of BRAF mutations. Next generation sequencing (NGS) has been shown to have high accuracy and sensitivity in simultaneously analyzing cancer-associated genes including BRAF. Together with dPCR these methods have been proposed as possible techniques for detection of mutations in FNA and liquid biopsy [46,47]. In the few studies comparing the two methods, the results reveal a high concordance in mutation detection between NGS and dPCR [48]. Even though results of direct comparison of dPCR and NGS in BRAF detection are not available in thyroid cancer it seems that dPCR can have a lower limit of detection compared to NGS in case of KRAS mutation [49].

Immunohistochemistry (IHC) has been suggested as a valid cost-effective alternative to genetic testing for the detection of BRAF mutation. The results rely mainly on the staining intensity and location and despite good concordance has been shown between positive strong BRAF protein staining and presence of BRAF mutation the results remain ambiguous in case of moderate and faint staining [50]. The concordance between diagnoses obtained by IHC and molecular methods is overall good and varies from 70% to 100% depending on variables such as the IHC protocol used and the sensitivity of the molecular method [51,52].

Detection of *BRAFV600E* mutation for diagnosis of thyroid cancer is well documented, however studies not always concord regarding the utility of *BRAF* detection for prognosis and survival of patient with thyroid cancer. It has been demonstrated that *BRAFV600E* and *TERT* promoter mutation each alone had a similarly modest effect while coexisting mutations exhibited a strongly synergistic effect on PTC-related mortality with a high HR of 37.77 in all PTC. Even more robust mortality associations of the genetic duet were seen when only conventional-variant PTC (CPTC) was analyzed (HR, 54.46; 95% CI, 12.26–241.82), which remained strongly significant (HR, 18.56; 95% CI, 2.97–116.18) after adjustment for clinico-pathological factors [39]. Remarkably, virtually no death occurred in patients harboring neither mutation while, in contrast, PTC-related deaths exclusively occurred with the coexisting *BRAFV600E* and *TERT* promoter mutations, suggesting that this genetic duet is a primary genetic mechanism for PTC-related mortality.

We assessed the utility of dPCR technique for identification of *TERTC228T* mutations in thyroid tumors. Consistent with previous data, *TERTC228T* mutations were not detected patients with FAs, FCs and MCs, but were identified in patients with PTCs. PTCs harboring both *BRAFV600E* and *TERTC228T* demonstrated morphological features of aggressive thyroid cancers, and one patient already developed distant metastases at the time of diagnosis.

Our results are consistent with previously published studies demonstrating that dPCR offers a clinically feasible platform that is relatively easy to use, with a quick turnaround time and, most importantly, high sensitivity [53]. Certainly, there are several caveats that would need to be addressed before the dPCR can be widely used in clinical practice. Despite the assay’s high sensitivity, attention should be given to collecting as much DNA as possible so as to increase the yield, especially in cases like liquid biopsies [54]. Another limitation of the conventional dPCR is the inability to detect more than one single mutation per assay. The methodology for dPCR is continuously improving, and we expect that in the future it can be used as a conventional diagnostic clinical tool. The emergence of multiplexing for dPCR is certainly encouraging [55], however in clinical practice a NGS model with identification of mutations for multiple genes of interest is certainly an attractive alternative.

It has to be noted that in contrast to NGS which can be a lengthy process, dPCR’s turnaround time is less than 24 hours, that can be particularly helpful in the cases of aggressive malignancies where time is of the essence [56]. In addition, cost for dPCR is significantly lower than NGS. The calculated costs of consumable for dPCR were also lower as compared to the cost of reagents for Sanger sequencing. The QuantStudio™ 3D Digital PCR System allows analysis of 24 samples, and the calculated cost of reagents is $10 per sample. In a recent medicoeconomic evaluation in the United States, the cost of sequential tests for *KRAS*, *EGFR* (*epidermal growth factor receptor*), *ALK* (*ALK receptor tyrosine kinase*), *ROS1* (*ROS proto-oncogene 1, receptor tyrosine kinase*), and *BRAF* was $3,763 ($464, $696, $1,070, $1,127, and $406 respectively), whereas the cost using NGS was $2,860 [57]. The cost of thyroid specific molecular test Thyroseq^®^ V2 is $3,200. The cost of Afirma^®^ BRAF test is $475 [58].

In summary, microfluidic digital PCR allows specific, sensitive and rapid detection of *BRAF*V600E and *TERTC228T* mutations in thyroid tissue samples and can be used for quantification of mutant alleles in thyroid tumors. This technique is easy to perform and economically competitive. Thyroid cancers with *BRAF* and *TERT* tend to have more aggressive phenotypes and often become resistant to traditional therapies. Thus, identification of these mutations in thyroid tumors offer a possibility for the use of therapeutic agents that selectively target oncogenic *BRAF*. Moreover, because of its high sensitivity, it could be also used for the analysis of circulating DNA for patients’ follow-up during treatment.

## 4. Material and Methods

### 4.1. Human Tumor Specimens

Thyroid tissue samples were obtained from an archival thyroid tissue bank maintained at the Department of Pediatrics of the Uniformed Services University of the Health Sciences. The study was conducted in accordance with the Declaration of Helsinki, and the protocol was approved by the Institutional Review Board Ethics Committee of Uniformed Service University of the Health Sciences (Project identification code: FG8661-S14)

The cases of this series were not selected for tissue quantity and quality, and consisted of 110 routinely processed formalin fixed paraffin embedded blocks from thyroid tumors operated in 1995–2003. These tissues samples were maintained in the tissue bank at the Uniformed Service University of the Health Sciences (USUHS). Initially, this set of samples was collected for the project “Molecular mechanisms of thyroid cancer invasion and metastases”, and, therefore, included thyroid cancers presenting with metastases at the time of surgery. Thyroid tissue samples were sectioned and histological diagnoses were established according to the WHO classification after examination of hematoxylin and eosin stained slides. There were 10 follicular adenomas (FAs), 10 follicular carcinomas (FCs), 5 medullary carcinomas (MC), and 75 papillary carcinomas (PCs), including 59 classical PTC (CPTC) and 16 cases of follicular variant PTC (FVPC). In 23 PTC cases, the central and invasive areas of tumors were identified using microscopy and micro-dissected for further analysis. We also examined lymph node metastases (LNMs) removed at the time of surgery from 22 patients.

### 4.2. Thyroid Cancer Cell Lines

Human thyroid cancer cell lines derived from follicular (FTC133) and papillary (BCPAP and TPC1) cancers were obtained from Dr. Motoyasu Saji (The Ohio State University, Columbus, OH, USA) with permission from the researchers who originally established the cell lines. All thyroid cancer cell lines had been tested and authenticated by short tandem repeat profiling analysis to be of thyroid origin. Cancer cells were propagated in conventional RPMI 1640 medium (Invitrogen, Carlsbad, CA, USA) supplemented with fetal bovine serum (FBS) to 10%, 100 U/mL penicillin, and 100 mg/mL streptomycin in a humidified 5% CO_2_ incubator. The cells were sub-cultured with 0.5% trypsin and 0.02% EDTA (Sigma-Aldrich, St. Louis, MO, USA) when the cell confluency reached 80%. All experiments were performed using thyroid cancer cell lines that had been passaged fewer than 20 times.

### 4.3. DNA/RNA Extraction

The Formalin-fixed paraffin-embedded (FFPE) of PTC samples were deparaffinized with xylene and rehydrated through ethanol (100%). The samples were digested with Proteinase K, incubated at 55 °C for 1 h with mild agitation and subsequently at 90 °C for an additional hour. Extraction of DNA and RNA from FFPE cancer samples were performed using King Fisher Duo Thermo Fisher (Thermo Fisher Scientific Inc., Waltham, MA, USA) according to the manufacturer’s instruction. The extracted DNA and RNA elute was stored at −80 °C. DNA and RNA quantification was performed by NanoDrop (Thermo Scientific, Inc., Waltham, MA, USA).

### 4.4. Sanger Sequencing

For Sanger sequencing, exon 15 of the *BRAF* gene was amplified by polymerase chain reaction using the primers 5′-CTACTGTTTCCTTTACTTACTACACCTCAGA-3′ (forward) and 5′-ATCCAGACAACTGTTCAAACTGATG-3′ (reverse). *TERT* promoter C228T and TERTC250T mutations were amplified using primers 5′-CAGCGCTGCCTGAAACTC-3′ (forward) and 5′-AGTGGATTCGCGGGCACAGA-3′ (reverse). The amplification protocol comprised initial denaturation at 94 °C for 2 min; 40 cycles of denaturation at 95 °C for 10 seconds, annealing at 55 °C for 30 s, and extension at 68 °C for 40 s; followed by a final extension at 68 °C for 7 min. The PCR product was cleaned with the gel filtration cartridges (Edge Biosystems, Gaithersburg, MD, USA). The sequencing reaction was performed with 100 ng of the purified PCR product with the above primers in both directions using the BigDye Terminator 3.1 cycle reaction kit (Applied Biosystems, Foster, CA, USA). DNA sequences and the BRAF /TERT mutation were determined using an ABI-PRISM 3500XI genetic analyzer (Applied Biosystems).

### 4.5. Microfluidic Digital PCR

3D Digital PCR analysis was performed on QuantStudio™ 3D Digital PCR System (Thermo Scientific, Inc., Waltham, MA, USA) using primers and probes for detection of *BRAFV600E*, *TERTC228T*, and *TERTC250T* (Thermo Fisher Scientific). The final 14.5 μL of reaction mixture contained 8.7 μL QuantStudio™ 3D Digital PCR Master Mix, 0.43 μL of primer/probe mix, 10 ng of DNA. The mixture was loaded into the QuantStudio™ 3D Digital PCR Chip. For *BRAFV600E* analysis chips were run on a ProFlex 2 × flat PCR System (Thermo Fisher) cycled with the following conditions: 1 cycle at 50 °C for 2 min; 1 cycle at 95 °C for 10 min; 45 cycles at 60 °C for 1 min and 95 °C for 15 s; 1 cycle at 60 °C for 1 min. For *TERT* promoter mutations, chips were run with the following conditions: 1 cycle at 50 °C for 2 min; 1 cycle at 95 °C for 10 min; 54 cycles at 55 °C for 1 min and 95 °C for 15 s; 1 cycle at 60 °C for 1 min. End point fluorescence data were collected on the QuantStudio™ 3D Digital PCR instrument and analyzed using the QuantStudio 3D AnalysisSuite software (Thermo Scientific, Inc., Waltham, MA, USA).

### 4.6. Statistical Analysis

Data were summarized by using frequencies and percentages for categorical variables and means and SDs for continuous variables. Shapiro-Wilk’s normality test was performed to access normal distribution. Student’s *t*-test for independent samples was used in continuous parametric variables and the chi-square test for categorical variables. In case of non-normal distribution Mann-Whitney test was used for analysis of two categories or Kruskal-Wallis test in case of more than two groups to compare. *p* values of 0.05 or lower were considered to be statistically significant. Pearson or Spearman correlations were used depending on whether the variable has a normal distribution or not. The SPSS statistical software version 22.0, (IBM Corp., Armonk, NY, USA) was used for all analyses.

## 5. Conclusions

Digital PCR is an advantageous technique for detection of *BRAF* and *TERT* mutations with high sensitivity, low time demands, and low costs. This technique provides an opportunity for quick and accurate detection of druggable targets in patients with thyroid cancer, and selection of appropriate treatment strategy. Implementation of dPCR in routine clinical practice could support a molecular-based stratification of prognosis in patients with thyroid cancer.

## Figures and Tables

**Table 1:** Analysis of BRAFV600 (B-type Raf kinase) mutation in function of clinico-pathological characteristics.

Clinical Characteristics	BRAF Positive (*n* = 42)	BRAF Negative (*n* = 33)	*p* Value
Age (mean ± SD)	38.64 ± 16	38.91 ± 14.7	0.996
Size (cm) (mean ± SD)	2.02 ± 1.3	2.1 ± 1.3	0.523
Histology FVPTC *	7.1%	39.3%	0.001
Gender male	19%	21.2%	0.816
Multifocality	42.8%	33.3%	0.401
Presence of invasion	48.7%	25%	0.048
Presence of lymph node metastasis	75%	35.4%	0.001

* Follicular variant papillary thyroid cancer.

**Table 2:** BRAF quantitative analysis related to pathological characteristics.

Pathological Characteristics	BRAF %	
	Mean ± SD	*p* Value
Central tumor with positive lymph nodes metastasis	27.65 ± 11.5	0.03
Central tumor without lymph nodes metastasis	18.4 ± 12.2	
Multifocality	26.83 ± 11.8	0.68
Non multifocality	25.2 ± 12.9	
Invasion	24.04 ± 14.6	0.34
Non invasion	27.79 ± 9.8	

## Disclaimer

The views expressed in this article are those of the authors and do not reflect the official policy or position of the United States Air Force, the Department of Defense, or the U.S. government. Title 17 U.S.C. 105 provides that “copyright protection under this title is not available for any work of the United States Government”. Title 17 U.S.C. 101 defines a United States government work as “a work prepared by a military service member or employee of the United States government as part of that person’s official duties”. This work was prepared as part of the official duties of Drs. Jensen and Vasko, Mr. Costello, Ms. Patel, and Ms. Mendonca-Torres.
